# Design choices for small-scale phase II trials with non-inferiority (NI) intention

**DOI:** 10.1186/1745-6215-12-S1-A89

**Published:** 2011-12-13

**Authors:** Hong Sun, Shu-Fang Hsu Schmitz

**Affiliations:** 1 Swiss Group for Clinical Cancer Research (SAKK

## Background

So far, NI trials are almost exclusively performed in phase III setting. However, the NI question might already be relevant in phase II setting in several scenarios, like: treatment optimization in diseases for which standard treatments already exist, or investigation of cytostatic agents, or rare diseases for which a phase III trial will not be feasible, or try to establish a reduced dose for frail patients. Before pursuing a phase III NI trial, a phase II trial is warranted. If resources are limited, then the scale of the phase II trials might be constrained. Feasible design choices for small-scale phase II trial with NI intention are desired.

## Methods

Both frequentist hypothesis testing and confidence interval (C.I.) approaches are considered for the intended NI phase II trials. Both single-arm and two-arm trials with a binary endpoint were performed, sample sizes obtained from the two approaches are compared under different parameter settings with the NI margin in the hypothesis testing approach equal to the interval width in the C.I. approach.

## Results

If the success rate of standard treatment is 0.5, with the NI margin 0.1 for the new treatment, the required sample size for one-sided hypothesis testing approach is at least 283 patients in two-arm trial with power 80% and 20% type I error (alpha) and 158 in single-arm trial with same power and 5% alpha. Using one-sided C.I. approach with 95% confidence level and allowed width of 0.1, only 76 patients are needed. The smaller the margin or the width is, the larger sample size is needed for either approach. The sample size from the C.I. approach is always lower than that from the other approach under the same settings.

## Discussions

The approach of the hypothesis test here is basically the same as for phase III trials, but could have a larger NI margin or alpha [1,2]. However, the determination of the NI margin is often problematic in a phase III setting and even more difficult in phase II setting as reliable previous data about the efficacy may not be available at this early stage. The C.I. approach does not require the NI margin or impose a rigid go/no-go decision rule only based on the primary endpoint. This allows the investigators to consider other aspects of the treatment for the final decision, hence might be preferred.
